# Co-designing a framework of the influences for the implementation of whole school approaches to Physical Education, School Sport and Physical Activity (PESSPA) in UK primary schools: development of the PESSPA Improvement Framework (PIF)

**DOI:** 10.1186/s12889-026-28832-w

**Published:** 2026-08-07

**Authors:** G. Jones, S. Kent, K. Longbon, S. Williams

**Affiliations:** 1 Advanced Wellbeing Research Centre, School of Sport and Physical Activity, Sheffield Hallam University, Sheffield, UK; 2 Physical Education and Sport Coaching, School of Sport and Physical Activity, Sheffield Hallam University, Sheffield, UK; 3 Sheffield Institute of Education, Sheffield Hallam University, Sheffield, UK

## Abstract

**Background:**

Reducing sedentary behaviour and achieving 60 min or more of moderate-to-vigorous physical activity daily provides physical and mental health benefits for children, however recommendations are not typically met. Whole-school approaches (WSA) are suggested to support children achieve these guidelines and emphasise the importance of multiple layers of a system working together to create change. Influencing factors over the implementation of WSAs to children’s physical activity provision is, however, still an emerging field.

**Methods:**

Regional multi-stakeholder exploratory workshops and interviews, and an online survey explored the development of an early framework that described influences of WSAs to physical education, school sport and physical activity (PESSPA) in UK primary schools. The double diamond design approach was employed as the underpinning design tool alongside a participatory research design using co-design. Three stages of data collection took place; (1) interviews and workshops to explore barriers and enablers to WSAs, (2) repeated the same processes, however identified and refined a working framework, and (3) employed an online survey to provide final framework refinement. Framework analysis was used during analysis. Framework layers and constructs were analysed in relation to the Theoretical domains framework (TDF) to aid understanding of key influences uncovered within the framework.

**Results:**

Participants identified a range of interrelated influences over the implementation of WSAs to PESSPA across multiple layers of a system. Central influences included senior leadership team (SLT) values and ethos, trust between stakeholders, school mindset, and school alignment of PESSPA in wider school priorities. Breadth and depth of school relationships and perceived opportunities and threats to PESSPA provision were highlighted as moderators, alongside circumstantial influences such as cultural and physical barriers and facilitators. Local, regional and national policy were also emphasised as key drivers to the implementation of WSAs to PESSPA provision. These findings informed the co-design of the PESSPA Improvement Framework (PIF).

**Conclusion:**

The PIF describes an initial, theory informed framework, highlighting key constructs that may impact the implementation of a WSA to PESSPA in UK primary schools. The PIF emphasises the importance of a multi-layered systems approach, working concurrently across PIF layers, to tackle physical inactivity in UK primary schools through the adoption of WSAs to PESSPA. TDF analysis highlights emerging influences over the implementation, or not, of a WSA to PESSPA, providing an early framework for targeted effort to improve the uptake of WSAs to PESSPA. In addition, future intervention design should focus on factors up-stream of local implementation, and how such interventions cascade down to a local level. Future research should build upon the PIF through collaboration with additional regional inputs as well as investigate causal relationships and established interactions of constructs between PIF layers.

**Supplementary Information:**

The online version contains supplementary material available at https://doi.org/10.1186/s12889-026-28832-w.

## Background

Physical activity (PA) is defined by the World Health Organization (WHO) as energy expenditure produced by the skeletal muscles [1] and ranges along a spectrum including sedentary behaviour, light, moderate and vigorous PA behaviour [2]. Research demonstrates that reducing periods of sedentary behaviour and achieving at least 60 min of moderate-to-vigorous physical activity (MVPA) each day leads to significant physical and mental health benefits for children [3]. Although links between the benefits of living a physically active life are understood, including prevention of chronic disease [4] and childhood obesity [4, 5], as well as improved physical, mental, social health [6–8] and academic performance [9–11], many children in the UK, and globally, do not meet these guidelines [12–14]. Children spend a significant amount of time within school, and thus this context is often an area targeted to support children achieve recommended PA guidelines [15].

Schools have historically employed simple, isolated interventions to date, which evidence suggests, are unlikely to have a meaningful, long-term and sustainable impact on PA outcomes as they occur within complex environments with many competing priorities [16–18]. Barriers to implementation, such as perceived priority of a policy, low staff confidence and capability, insufficient time, timetabling, financial support, perceived detrimental effect on learning time, competing priorities, and contextual characteristics (e.g., physical environment) [19–23] are often cited. Consequently, Public Health England and the WHO have recommended that schools adopt whole-of-school approaches (WSA) to PA. WSAs emphasise the importance of a multi-layered approach across a *system*, and recognises that a system does not work in isolation. Thus, a WSA goes beyond individual intervention, such as policy updates [24], and instead, emphasises the operationalisation of collective initiatives [25, 26]. A WSA focuses on contextual awareness, collaborative practices, understanding power dynamics across stakeholders, and non-linearity and complexity of both the issue and the solution [27–29], thus continue to evolve and adapt to an everchanging context. WSAs, therefore, demonstrate a promising route forward [26, 30] given the complex nature of the school environment and implementation of Physical Education, School Sport, Physical Activity (PESSPA) initiatives.

Within the UK, evidenced based WSAs to PESSPA are emerging [31–34]. Early evidence suggests WSAs are acceptable by school staff, however their feasibility is questioned [31]. This could be due to implementation issues such as workload and confidence [35, 36] rather than belief about their potential for change [21]. Measuring WSA ‘change’ is different to intervention effectiveness (e.g., PA behaviour) and relates to understanding complex contextual conditions [37] that are hypothesised to influence the target behaviour. Due to the complexity of WSAs, change may occur slowly, making it more difficult, and take longer to evidence impact, potentially due to interventions focusing on areas such as school culture. The issue, therefore, is not the absence of robust WSAs, but rather a growing recognition for the difficulty of implementing WSAs over time [31], likely resulting in interventions that remain isolated, uncoordinated, and not delivered in a WSA manner. Further clarification and identification of mechanisms that influence a school’s implementation of a WSA to PESSPA, that work across intersecting micro-meso-macro layers would greatly benefit the literature.

A growing trend to explore and understand the context in which a school sits, is becoming more evident [25]. This is because there is a greater appreciation for how an intervention is, or is not, likely to be effective based on variances in the context in which an intervention is placed. Contextual examples include, but are not limited to, the physical and cultural environment, economic position, geography, past behaviour and experiences, attitudinal and normative beliefs, and local and national politics [38, 39]. When a given context is understood, relevant intervention influences can then be implemented to create change. To date however, a holistic picture of the vast influences at play within a school’s context which can influence the implementation of a WSA to PESSPA are not fully understood. It is important to comprehend the web of these influences due to the variance in contexts in which schools sit within (e.g., socioeconomic status and the built environment). A recent systematic review [40] highlighted key barriers and facilitators for health behaviour interventions in school settings, finding senior leadership team (SLT) values, accessibility of the built environment and/or equipment, and school staff team cohesion all had important roles over the decision to implement, or not, health related interventions. This review was not specific to PA, nor did it focus on primary schools. Although this review did highlight exploratory research had been conducted under the topic of health behaviours in schools, few studies have attempted to gain a holistic picture of the varying influences over the implementation of WSAs to PESSPA in UK primary schools [31, 32], thus further exploration of this topic would aid the literature.

Health behaviour researchers have advanced knowledge of predictive models of behaviour [41] and draw upon robust intervention development frameworks such as the Theoretical Domains Framework (TDF) [42, 43] and the Behaviour Change Wheel (BCW) [44, 45]. The BCW goes beyond the scope of this study as it focuses on intervention design, however interpreting the results in line with the TDF is valuable, as it enables the identification of potential key domains. Previous studies employing the TDF under this topic have highlighted early domains for intervention within a school (e.g., knowledge, goals, leadership) [46] and when investigating how policy influences practice (e.g., environmental context and resources, goals, knowledge, intentions, and social influences) [20, 47], however findings tend to orientate around micro-meso-level based strategies (i.e., within school). The TDF synthesises numerous behaviour change theories into a comprehensive framework of 14 domains, which include knowledge, skills, social/professional role and identity, beliefs about capabilities, optimism, beliefs about consequences, reinforcement, intentions, goals, memory, environmental context and resources, social influences, emotions, and behavioural regulation. These domains help the identification of influencing factors on a behaviour [42].

The aims of this study were to;

- Co-design an early framework describing the influencing factors over the implementation of a WSA to PESSPA in UK primary schools
- Identify critical influences over the implementation of WSAs to PESSPA
- Relay PESSPA Improvement Framework (PIF) data over the TDF to determine emerging TDF domains.

## Methods

A participatory research co-design process was employed. This enabled a collaborative approach whereby relevant stakeholders were actively involved throughout the research process to co-generate and refine outputs, ensuring that any resultant output/framework was grounded in real-world experience and reflected the complexities of attempting to implement a WSA to PESSPA in UK primary schools.

### Participants

Participants were recruited via convenience sampling, due to the demand required of participants. Recruitment utilised regional (South Yorkshire) networks advertising the research via email distribution through authors networks. Participants were required to work in the education sector, or have influence over the education sector (e.g., public health). Seventeen people responded to the expression of interest. Those who responded to the call were forwarded a participant information sheet and consent form. Eight people agreed to participate and provided written informed consent. Those who did not provide informed consent were excluded from further involvement in the study.

Eight participants (female: *n* = 4, male: *n* = 4) were involved in the development of the PESSPA Improvement Framework, all of whom were known to the research team. Participants represented various school stakeholder groups, including primary school SLT (*n* = 1), school PE leads (*n* = 3), regional sport foundation (*n* = 1), local authority public health team (*n* = 2), and local authority strategic leadership partnership (*n* = 1).

### Procedure

**Fig. 1:**
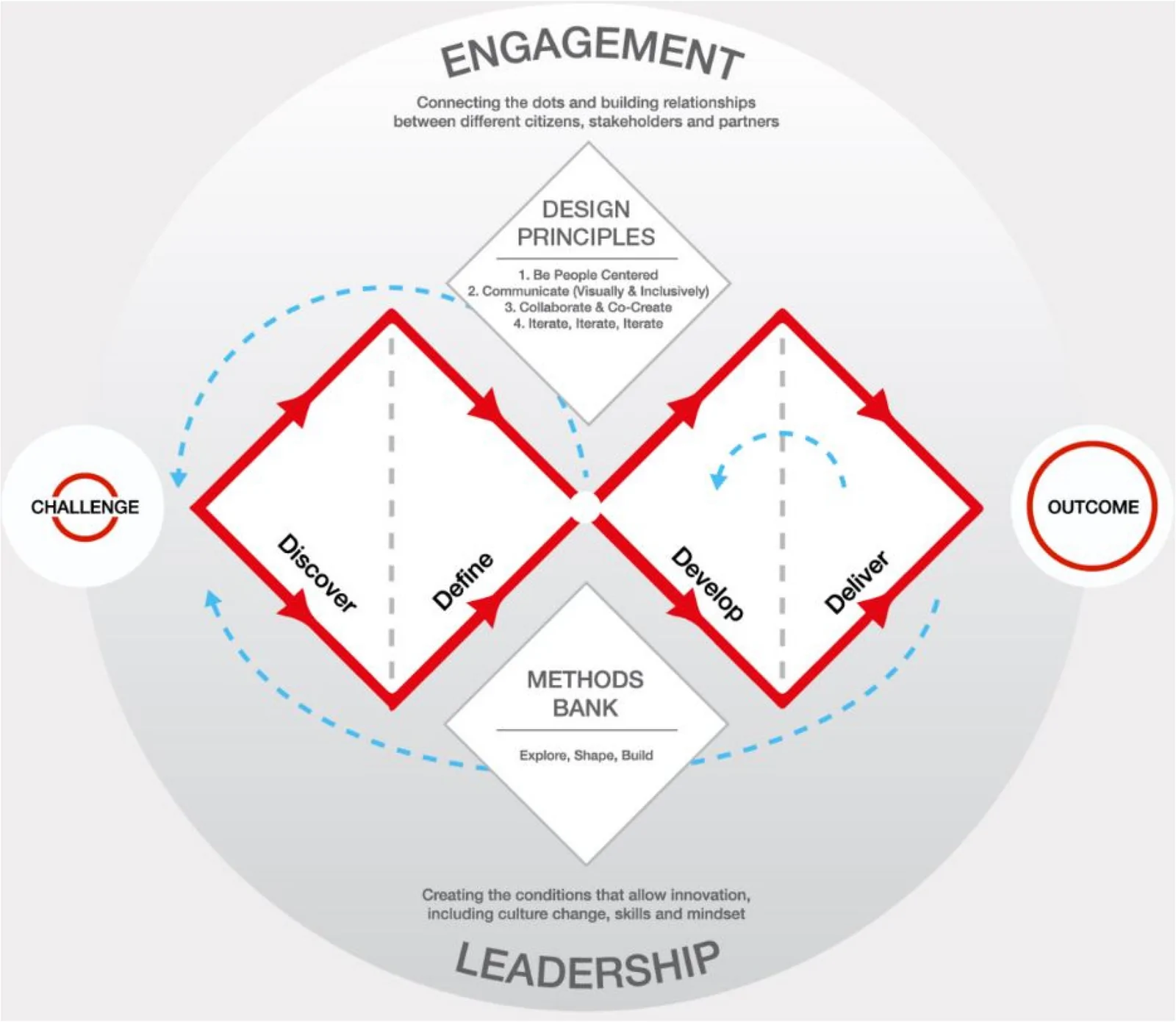
The Double Diamond Design Approach (DDDA) [49]

**Fig. 2:**
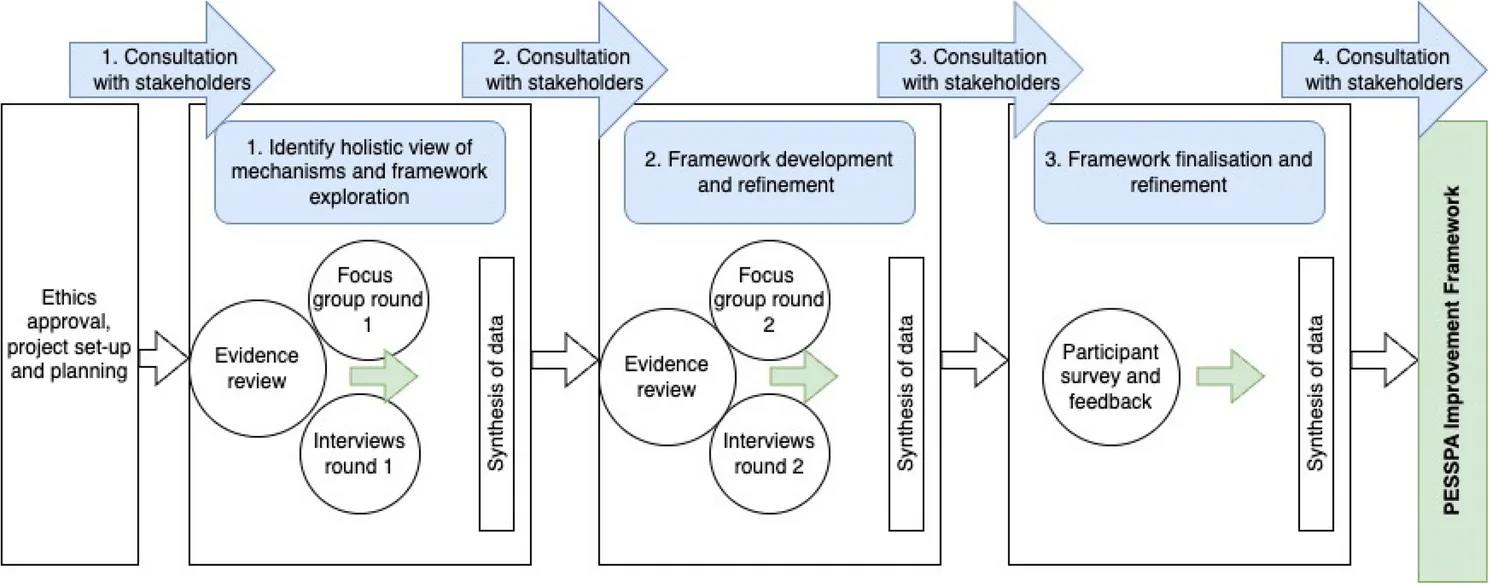
Framework of the iterative PIF design process

The exploratory nature of this research required a qualitative approach to gain rich and relevant data appropriate to address the research aims, to co-design a framework of the influences for the implementation, or not, of a WSA to PESSPA. Co-design was selected to develop the initial framework and to be refined through co-production in future studies. The consolidated criteria for reporting qualitative research (COREQ) 32-item checklist [48] was utilised to improve reporting. Data collection took place via focus groups, interviews and an online survey, conducted by authors GJ, SW, KL, SK. The double diamond design approach (DDDA) [49] was utilised to promote divergent thinking and capture a holistic overview of ideas, challenge thinking, and support convergence of ideas for refinement and creating a succinct framework. This followed a process of discovery, definition (Stage one), development (Stage two) and delivery (Stage three) (see Figs. 1 and 2). Fig. 2 represents the iterative data collection process across the three stages of framework development. In total, two focus groups and nine interviews were conducted between November 2023 and July 2024. The questionnaire was used post focus groups and interviews to provide final framework feedback. All participants were involved in either the workshops or interviews at both stages one and two of framework design. All focus groups and interviews were audio recorded and transcribed verbatim. Lead author (GJ) has significant knowledge and expertise in conducting and analysing qualitative data. 

Semi structured focus groups and interviews (Table 1) were conducted based on both an inductive approach, where participants were asked to generate themes and ideas, and a deductive approach where a literature review yielded initial prompts for discussion (e.g., barriers and facilitators to PESSPA) and used during stage one focus group (*n* = 4) and interviews (*n* = 4). There was an emphasis on the inductive process, with critique of deductive prompts used to mitigate bias. A complete interview guide can be found in supplementary material. All focus groups and interviews were conducted in person, and participants were able to write down their ideas on post-it notes and used A0 paper to record ideas. Attending researchers facilitated this process, recording themes and ideas to allow for group discussion to flow. Wherever a researcher did this, clarification was sought to understand if the researcher interpreted what was being discussed accurately. Participants, through discussion, agreed, clarified, moved, or disregarded influences and/or themes. Photographs were taken of data written on flipchart paper for analysis purposes. Following workshop one and interviews, authors digested and synthesised data and utilised framework analysis [50] to generate a first draft of the framework with lead author analysing all transcripts, and authors SW and KL analysing a subset of transcripts. All authors met to discuss data codes and layers/constructs generated independently until a consensus of data interpretation was met to add rigour to the process. During stage two of the process, a second focus group (*n* = 2) and set of interviews (*n* = 6) were conducted (Table 1), where participants critiqued the initial design of the framework further (see Fig. 2). Authors interpreted stage two focus group and interview data as they did for stage one, to produce an updated version of the PESSPA Improvement Framework for stage three. Focus groups one and two lasted one hour 24 min and one hour and six minutes respectively. Interviews lasted an average of 55.10 min (SD 12.65 min). Stage three, an online questionnaire, presented the latest version of the PIF and asked participants to offer final amendments, which were all included into the published version. Five participants responded and completed the online questionnaire to revise or amend the final version of the PIF.

**Table 1:** Topic guide example questions

Stage one Topic	Stage one Questions
Understanding of whole school approach	What does a whole school approach look like?
Exploring whole school approach influences	Jane is a primary school teacher who recently moved to a school with no WSA to PA. What might Jane encounter when trying to implement a WSA to PA? What are the root causes of these?
Crucial factors	What are the most significant hurdles Jane will need to overcome to be able to begin to implement a WSA to PA in her new primary school? What are the most significant facilitators Jane could have at her disposal to begin to implement a WSA to PA in her new primary school?

**Stage two Topic**	**Stage two Example Questions** ***(Present preliminary results based on Stage one)***
Check interpretation	How well might the items, themes and feel of the framework, represent what Jane may encounter when trying to implement a WSA to PA in reality? Discuss if you think the crucial factors that Jane may encounter are highlighted.
Crucial factors	Discuss what are the most significant (top 3) influences that will influence Jane in her quest to implement a WSA to PA in her new school.

**Stage three Survey Topic**	**Stage three Survey Example Questions**
Diagram	If there are any themes or constructs of the diagram that you do not agree with, think need moving or re-wording, or if there are themes or constructs you believe are missing and need adding, please highlight / detail all of these here.
Table	If there are any items within the table that you do not agree with, think need moving or re-wording, or if there are items you believe are missing and need adding, please highlight / detail all of these here.
Final thoughts	Please provide any final thoughts and opinions of the PESSPA Improvement Framework here

### Data analysis

Framework analysis [50] was utilised to analyse data. This includes seven stages; (1) transcription, (2) familiarisation, (3) coding, (4) developing a working analytical framework, (5) applying the analytical framework, (6) charting data into the framework matrix, and (7) interpreting the data.

Final analysis then saw the PIF being analysed using the qualitative data to interpret emerging influential constructs within the PIF (Table 2), where salience/strength of the construct, consistency across participants about the item/construct/layer, and any presence of conflict (i.e., a participant disagreed with the construct/item, proposing an alternative) were used to interpret the data. PIF constructs were then mapped onto the TDF for interpretation of potential future intervention functions [42, 43] (Table 3). Finally, the top three influential PIF constructs, as voted for by participants in stage two workshop and interviews, were presented (Table 4).

**Table 2:** Identification of emerging key PIF constructs

PIF Layer	PIF Construct	Supporting quote
Core principle	**Leadership team values and ethos**	“*The leadership quality. What is it*,* how they live*,* and model themselves or how they influence everyone around them*,* to get on the bus*.” (Participant: Local authority public health lead)
	**Trust between stakeholders**	“*SLT and the head. My head was an ex-PE lead so he values what I do and that’s why I’m in the role and he’s created this role and I’m one of the only full-time PE staff*.” (Participant: PE Lead)
	Mindset of school	Not applciable
	Alignment across school	Not applciable
Moderators	Opportunities and risks	Not applciable
	**Relationships**	“*Because although you need a really strong PE lead who’s going to sort of drive it forward… you do need your staff*,* all your staff*,* to be competent*,* teaching PE…They can’t be in every lesson and seeing what’s going on… I’ve just started working for a sports partnership one day a week*,* and I go in and mentor primary school teachers in their classes*,* like in a process like over a term or whatever amount of time and we like look at targets and go into their lessons and we just improve their quality of their teaching*.” (Participant: PE Lead)
Circumstantial influences	Physical barriers and facilitators	Not applciable
	**Cultural barriers and facilitators**	“*I think a head teacher that takes risks*,* and that can be something very*,* very minute in terms of*,* we want to transform our play provision… You know it’s well*,* if the head teacher is willing to take risks and actually find solutions rather than reasons to not do something*,* we’re seeing significant change when that’s the approach of the head teacher. So it could be from that aspect*,* but also strategically. With the other pressures that the head teachers are getting*,* whether they’re in the Ofsted window or you know whatever it might be. Still ploughing ahead and saying no*,* we’re going to do this despite all of that.*” (Participant: Regional sport foundation)
	**School values (Recognised relevance)**	“*Definitely leadership… What’s the SLT values on this new scheme of work… The alignment between the stakeholders*,* probably*,* yeah… I like it to be evidence based. I like reading up and making sure I don’t just dive in. Whole school approaches on anything. Right*,* OK*,* what evidence is there? Let me read some reviews. Let me read the research based on what they’ve done*.” (Participant: Head Teacher)
	WSA characteristics (Recognised relevance)	Not applciable
Strategic agenda	National priorities	Not applciable
	Local authority priorities	Not applciable
	Multi-academy trust priorities	Not applciable
	**Senior leadership team priorities**	“*The deputy that stepped up*,* who has been very much part of that culture. That opportunity of active enrichment has continued seamlessly despite these changes that have been made… So it probably comes back to that* [SLT priorities] *because that’s just so ingrained and solid*.” (Participant: Regional sport foundation)

PIF constructs without supporting quotes were not emphasised in the data as potentially key influences

**Table 3:** Emerging key TDF domains

#	TDF domain	Supporting quotes for emerging key TDF domains
1	**Knowledge**	“*The PE leads got to be knowledgeable. If you’ve got SLT involved*,* they’ll prioritise. For those opportunities*,* they’ll put finances around it. They’ll give dedicated time to a PE lead*,* which will then enable that if they’ve got the right PE lead*,* enable it to happen*.” (Participant: Local authority education partnership)
2	**Skills**	“*Get somebody with expertise and skills to come along and show them*,* you know*,* we do a staff meeting where somebody shows them five examples of that. Build physical activity into your teaching and maybe accompanies that with some science to explain what happens to the brain. What happens to why that? Why does that help? Well*,* so you’re doing a bit of explaining why it matters and then you show them some ways of doing it and quite a lot of people will just go*,* OK*,* get that. I understand why it’s important*.” (Participant: Local authority education partnership) “*Emphasis on that physical activity*,* whether it’s PE curriculum*,* CPD*,* or just*,* you know*,* looking at how we can train up our lunch time staff so that they’re not sort of standing in corners*,* just chatting to each other*,* but they’re actually promoting physical activity*,* but that has to come from the top to make time for those staff to attend that training*”. (Participant: PE lead)
3	**Social / professional role and identity**	“*If you’ve got a head teacher who doesn’t value physical sport or PE*,* then those opportunities don’t happen*” (Participant: Head teacher) “*I think that’s the point* [strong school cultural identity/values] *where you start unlocking it as whole school* [approach] *because it sort of at that point you’re then looking for. I think right this is a priority. I’m open to what does doing this well look like?*” (Participant: Local authority education partnership)
4	**Beliefs about capability**	“S*o effectively staff understanding and valuing PE… is the ultimate game or the ultimate aim… they obviously have a full time PE lead which a lot of them are grateful for. I obviously offered CPD to them and help with ideas and effectively reduce their cognitive load at times depending on the staff member depends on how comfortable they are and again that’s from background and how they have received PE in the past or the experiences in the past… whilst some female staff aren’t as confident in teaching games I’ve got some dance specialists now or some gymnastics specialist I’ve been able to send on further training and things like that so there’s a bit of a balance which is good… the teachers sometimes will need my help delivering stuff or they’re fully competent and actually they don’t need me at all it’s just their area and their background*” (Participant: PE lead)
5	Optimism	Not applciable
6	Beliefs about consequences	Not applciable
7	Reinforcement	Not applciable
8	**Intentions**	“W*hole school approach to PA is all staff understanding the benefit for the children. Effectively it is obviously the PE lead who will drive it and I’m lucky in some respects I’m a full time PE lead because I know obviously the pressures from part time stuff that it has to be sorted from the top down the culture of the school has to be asked to come in from SLT as well backing up the PE lead. Because if the PE lead sets things in place but then things are being pulled by other teachers and you don’t have that backing from your head or from deputy heads or whoever else then everything falls flat on its face and PE becomes ostracised and sort of kept in this bubble*,* in this pocket*,* especially with the pressures of SATs and the core subjects as they would be called maths*,* science*,* English be taken the lead in schools*,* sort of quite lucky here that PE become one of these sort of core subjects as well that’s valued and my role is valued as well.*” (Participant: PE lead)
9	**Goals**	“*If you can convince them* [school staff] *that it* [PESSPA] *supports those wider priorities and leavers* [goals], *then it it’s much more likely to be* [prioritised] *… if that one’s in place*,* a lot of those will follow. Because that’s*,* a way in which you might establish relative importance*.” (Participant: Local authority education partnership) “*If you had a head teacher or the school values of the school did not really believe in PESSPA. Coming along with the whole school approach to this activity*,* it’s probably not gonna be implemented*,* whereas if it aligns or they can understand how PESSPA aligns with their school values*,* then so like life skills and it’s about this kind of embracing change*.” (Participant: Regional sport foundation)
10	Memory, attention and decision processes	Not applciable
11	Environmental context and resources	Not applciable
12	**Social influences**	“*We have a couple of teacher races just to show*,* actually adults can do this as well. So you know*,* we model… we model it*,* we also run and yeah and jump and do things*.” (Participant: Head teacher) “*Without SLT and the backing of my head nothing can get done. They’re* [staff] *as vital as everybody else*,* so then again their experiences with PE and how they* [SLT] *value PE feeds down and becomes a culture of the school*.” (Participant: PE lead)
13	Emotion	Not applciable
14	Behavioural regulation	Not applciable

TDF domains without supporting quotes were not emphasised in the data

**Table 4:** Top 3 influential constructs cited in stage two workshop and interviews

PIF construct	Frequency of the most cited construct influences across workshops & interviews
SLT team values and ethos	**7**
Cultural barriers and facilitators	**6**
(SLT and PE lead quality)	(4)
(Staff buy in)	(1)
(Parent support)	(1)
Trust between stakeholders	2
Alignment across school	2
Recognised relevance (school values)	2
Relationship breadth and depth	1
National guidance	1

## Results

### Qualitative findings

Findings from Stage one workshop and interviews highlighted a range of interrelated emerging and potentially key themes across multiple layers of a system over the implementation of a WSA to PESSPA in UK primary schools. Influences included the role of SLT, school culture, physical barriers and facilitators, trust between different school employees, the overarching mindset and approach of a school to engage in PESSPA initiatives, the alignment of PESSPA provision within wider school priorities, and the role of national and local agenda. Participants discussed potentially key influences, including school leadership, trust between key staff members, both inter and intra school relationships, cultural and barriers and facilitators, overarching school values, and policy (Table 2).

### Development of the PESSPA Improvement Framework

Stage one provided enough data to create a first version of the PIF diagram and table, which included four layers. The most central layer was COM-B, second were core principles made up of national agenda, local authority priorities, trust, leadership quality, alignment and SLT values, third were moderators made up of relationship breadth and depth, risks and school mentality, fourth were barriers and facilitators made up of physical and cultural, and relative value (school values and WSA characteristics). Stage two refined the PIF diagram and amended the items in the PIF table, creating a five layered framework, similar to the final version presented. Stage three offered minor amendments to the PIF diagram, such as reordering the strategic agenda layer from the third to fifth layer, updated wording (e.g., ‘mentality’ to ‘mindset of school’) and adding MATs as an explicit note into the strategic agenda layer. Minor wording tweaks were also made to the PIF table. Five participants responded to the questionnaire and stated the ‘Layers’ and ‘Constructs’ of the PIF reflected reality ‘Extremely well’ (*n* = 1*)* or Very well’ (*n* = 4*)*. All participants stated the ‘Items’ (i.e., detailed descriptors making up a construct) and descriptors of the PIF reflected reality ‘Extremely well’ (*n* = 2*)* or ‘Very well’ (*n* = 3).

**Fig. 3:**
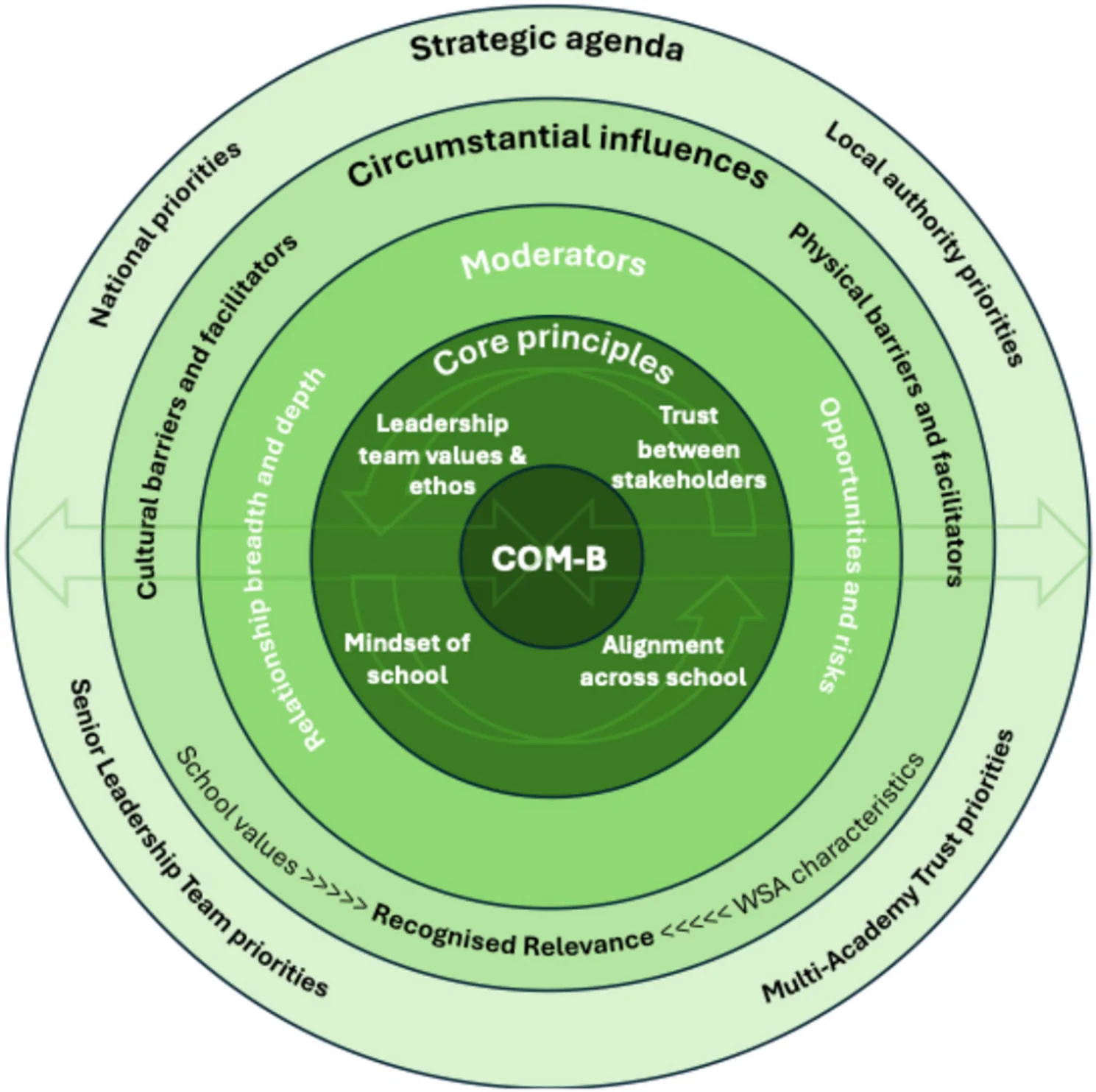
Diagram of the PIF layers and constructs

The final version of the PIF included five layers (Fig. 3 and Table 5). The most central layer of the PIF diagram was the COM-B model, as this was the theoretical underpinning to the framework design due to the intention of the PIF being to understand what can help or hinder behaviour to implement a WSA to PESSPA in UK primary education settings at an individual level. The other four layers included; core principles, moderators, circumstantial influences, and strategic agenda. Nested within these four layers were 12 constructs, including (1) core principles; leadership team values and ethos, trust between stakeholders, mindset of school, alignment across school, (2) moderators; relationship breadth and depth, opportunities and risks, (3) circumstantial influences; cultural and physical barriers and facilitators, recognised relevance which included school values and WSA characteristics as sub-constructs, and (4) strategic agenda; national priorities, local authority priorities, multi-academy trust priorities, and senior leadership team priorities. A detailed exploration of these layers and constructs is demonstrated in the PIF table (Table 5), highlighting the individual ‘items’ which support PIF constructs and layers. Table 6 demonstrates a working example of how raw data was translated into the framework. 

**Table 5:** PIF layers, constructs, and items

Layer	Construct	Item
All layers can positively or negatively influence these constructs	Capability	Physical Capability: Physical capability refers to whether an individual possesses the necessary knowledge and skills required to perform the target behaviour.
		Psychological Capability: Psychological capability refers to an individual’s capacity to engage in the necessary thought processes, comprehension, and reasoning to perform a target behaviour.
	Opportunity	Physical Opportunity**: **Physical opportunity is afforded by the built environment.
		Social Opportunity: Social opportunity is afforded by the cultural milieu that dictates the way that individuals think about things.
	Motivation	Reflective Motivation: Reflective motivational includes processes that involve evaluations and plans.
		Automatic Motivation: Automatic motivational included innate processes’ that involve emotions and impulses.
Core Principals	Leadership Team Values and Ethos	Quality and consistency of leadership throughout SLT, PE Lead, and any key people across the school, including knowledge, role modelling of behaviours / values, and strategic thinking.
	Trust between stakeholders	Trust between SLT, PE lead, staff, parents and pupils to take initiative and be provided autonomy to lead or support PESSPA provision.
	Mindset of school	The mindset of key personal (e.g., SLT & MAT) within a school environment to influence other school stakeholders (e.g., school staff). For example, being open to change, bold or brave new behaviours or ways of working. It is recognised that aspects of WSA's to PESSPA can be perceived as risky or innovative.
	Alignment across school	Alignment of values, future direction, and actions between leadership, staff, parents, and children.
Moderators	Opportunities and risks	Contextual features that offer an opportunity or risk towards implementation of a WSA to PESSPA, such as change of leadership, change of PE Lead, competing priorities, SLT or staff (non)buy in.
	Relationships	The breadth and depth of relationships between different stakeholders both internal and external to a school.
Circumstantial Influences	Barriers and facilitators	Cultural Barriers and Facilitators
		· Government / Ofsted / MAT perceived and actual support for PESSPA and WSAs
		· SLT priorities, knowledge and understanding of PESSPA and WSAs
		· PE lead knowledge and understanding of PESSPA and WSAs
		· PE lead confidence in supporting school needs for PESSPA and WSAs
		· Dedicated (i.e., time & knowledge resource) PE Lead
		· School staff buy in, confidence, skills, knowledge, and values for PESSPA
		· Support for school staff through CPD offer in relation to PESSPA
		· School staff health and wellbeing
		· Prioritisation for PESSPA time tabling, financial support, and opportunities (inter and intra)
		· External organisation support links (e.g., School Sport Partnerships, community clubs etc)
		· Influence of other schools priorities and actions
		· Children’s needs and buy in
		· Pupil voice and feedback on PESSPA initiatives
		· Curriculum & other schemes of work
		· Socioeconomic status of the immediate community
		· Readiness of the school to act on PESSPA initiatives
		· Parental attitudes towards PESSPA provision
		· Governor attitudes towards PESSPA provision
		Physical Barriers and Facilitators
		· School infrastructure (facilities)
		· School equipment
		· School finances
		· Community facilities (e.g., pools and parks)
		· School Sport Partnership's or similar, access
		· PESSPA capacity (resource, finance, training) available
		· Technology available in a school
	Recognised relevance	WSA characteristics: The introduction of a WSA in a school, depends on the demonstration of:
		· Support a school’s whole school improvement plan
		· Support a school’s wider priorities, aims and ambitions
		· Realistic implementation (e.g., time frame, looking to achieve, resourcing required)
		· Be flexible in application
		· Address’ inequalities in a school and have targeted activities embedded within
		· Be evidence based
		· Be used to demonstrate a positive impact on key school objectives (e.g., learning)
		School values: In order for a WSA to be introduced in a school, connections between the WSA and other school values need to be made. Consider:
		· Whole child enrichment
		· School physical literacy importance relative to other values
		· Opportunities for life skills to be developed
		· Being embedded throughout school
		· Physical activity being seen as a tool for teaching and learning
		· The connection between PESSPA and health and wellbeing
Strategic Agenda	National priorities	What are the national priorities of the government and how do these align with PESSPA provision and the use of whole school approaches?
	Local authority priorities	What are the priorities of the local authority and how do these align with PESSPA provision and the use of whole school approaches?
	Multi-Academy Trust priorities	What are the Multi-Academy Trust's priorities and how do these align with PESSPA provision and the use of whole school approaches? (NB: if applicable)
	Senior leadership team priorities	What are the immediate school SLT priorities, and how do these align with PESSPA provision and the use of whole school approaches?

**Table 6:** An example of how raw data informed the PIF

Quote	Code	Item and Construct	Framework layer
“*Money that came into a centralised pot… But head teachers didn’t have a clue about what to spend it on*,* and in some cases still don’t unless they’ve got a* [Knowledgeable PE lead] *who’s really good*,* and knows what to spend the money on*.” (Participant: Local authority public health lead)	Distributed leadership	Item: Trust between SLT and PE Lead Construct: Trust between stakeholders	Core Principles
“*I’m coming at it from the perspective of the knowledge of the communities that those schools. That are in the trust. Work with and some of the challenges that those communities that the schools face and trying to engage children and young people are doing physical activity. Yeah*,* some of which is culture*,* some of which is inequality*,* some of which is poverty*.” (Participant: Local authority public health lead) “*We teach it*,* but we’ve just made a link with* the [community provider]. *And* [community provider] *has really*,* really helped us out with what*,* just the amount that we’re paying for that*,* so Year 5 go in and do squash in their PE lessons*,* Year 6 go in and do tennis. So suddenly we’re going*,* oh*,* actually in* [local primary school] *are doing this*,* and we’re suddenly on that footing*,* paying not as much. I guess your links if you’ve got good links in the area that. Yeah*,* yeah*,* yeah.”* (Participant: PE lead)	Connection between stakeholders	Relationship breadth and depth	Moderators
*“I was chatting with* [head teacher] *recently and talking about our curriculum… And I’ve sort of built upon that and I’m coming back to teacher competence. When I was off through injury. A lot of teachers*,* we became the best dodgeball school in the in the country I’m sure*,* because that is all that they wanted to do and then they were like*,* well*,* we picked up the planning and it looks complicated. And again staff time comes back into that. But me and* [staff member] *both being PE leads figured out*,* we looked at it* [PE curriculum] *in a completely different way to how somebody else would pick it up*,* so kind of created a two page lesson plan that staff can just pick up*,* at the bottom of the second page is always a step box*,* just so they’ve got some ideas that they need. And staff have sort of been like oh*,* this has been brilliant*,* actually*,* we can follow this*.” (Participant: PE lead)	Knowledgeable PE lead.	Item: PE lead Knowledge and understanding of PESSPA and WSAs Construct: Cultural Barriers and Facilitators,	Circumstantial influences
“*I think one of the strongest drivers in schools around*,* which I’m looking at*,* is both leadership*,* in so far as the leadership I mean*,* a sort of willingness for the school value*,* which*,* because I think most schools*,* I certainly know for the* [multi academy] *trust that I’m part of*,* the values are clear that it is about children being healthy*,* well*,* and the recognition that physical activity is a really important part of their development*.” (Participant: Local authority public health lead) *“it’s about the local city… the Council’s identifying* [Local area] *city goals. These are*,* I think there’s 18 goals. It’s about transforming*,* transforming the city*,* basically. And physical activity and health is one of the goals… This is implementation of the* [local area] *city goals*,* local authority priorities… It identifies for school that what they are delivering if part of the city plan*.” (Participant: Local authority public health lead)	Inter Leadership values importance Wider system impact	MAT priorities Local priorities	Strategic Agenda

### Core principles

Four core components were highlighted to underpin the ways of working within a school environment. *Leadership team values and ethos* were thought to be front and centre with the quality and consistency of this leadership being key to long term success. Factors such as depth of knowledge, role modelling and implementation through a school strategy were vital. *Trust between stakeholders* were emphasised to enable staff other than SLT (e.g., PE lead or classroom teachers) the autonomy and belief that implementing PESSPA strategies into the school day was valued and rewarded. *Mindset of the school* were emphasised to come from key strategic personnel within the school environment, to have an influence to be ‘bold’ or ‘brave’ as some PESSPA initiatives within schools were perceived as ‘risky’ (e.g., active classrooms). Lastly, *alignment across school* was a pivotal principle and reflected the importance that an agreed school strategy has an underlying impact on the day-to-day implementation of a WSA to PESSPA.

### Moderators

Two moderating components were identified as having unique impacts on the core principles and circumstantial influences. The first, *relationships breadth and depth*, spoke to the strength and number of relationships both internal and external to the school. For example, increased quantity and meaningful relationships internal and external (e.g., local authority or School Games Organisers) a school’s PE lead had would aid the implementation of a WSA to PESSPA. The second, *opportunities and risks*, described how changes in the school structure or external influences could impact the implementation of a WSA to PESSPA. Key examples included change in school leadership or PE lead, national policy, and the presence of competing priorities across varying layers within a school.

### Circumstantial influences

Circumstantial influences identified three components, (1) *cultural barriers and facilitators*, (2) *physical barriers and facilitators*, and (3) *recognised relevance*, which contained sub-categories (a) *WSA characteristics* and (b) *school values*.

*Cultural & physical barriers and facilitators* were suggested to have the potential to impact positively or negatively, depending on the direction of change. 18 unique items were identified (see Table 5), (1) governmental perceived and actual support for PESSPA provision, (2) SLT priorities, knowledge and understanding of PESSPA and WSAs, (3) PE lead knowledge and understanding of PESSPA and WSAs, (4) PE lead confidence in supporting school needs for PESSPA and WSAs, (5) dedicated (i.e., time and knowledge resource) PE lead, (6) school staff buy in, confidence, skills, knowledge and values for PESSPA, (7) support for school staff through CPD offer in relation to PESSPA, (8) school staff health and wellbeing, (9) prioritisation for PESSPA time tabling, financial support and opportunities (inter and intra), (10) external organisation support links, (11) influence of other schools priorities and actions, (12) children’s needs and buy in, (13) pupil voice and feedback on PESSPA initiatives, (14) curriculum & other schemes of work, (15) socioeconomic status of the immediate community, (16) readiness of the school to act of PESSPA initiatives, (17) parental attitudes towards PESSPA provision, and finally, (18) governor attitudes towards PESSPA provision.

*Physical barriers and facilitators* focused on tangible influences on the ability to implement a WSA to PESSPA. Seven items were identified, including (1) school infrastructure (facilities), (2) school equipment, (3) school finances, (4) community facilities (e.g., pools and parks), (5) school sport partnerships or similar, access, (6) PESSPA capacity (resources, finance, training) available, and (7) technology available in a school.

*Recognised relevance* was divided into (a) *WSA characteristics* and (b) *school values*. WSA characteristics had seven items, including (1) support a school’s whole school improvement plan, (2) support a school’s wider priorities, aims and ambitions, (3) realistic implementation (e.g., time frame, achievement, resourcing), (4) be flexible in approach, (5) address’ inequalities in a school and have targeted activities embedded within, (6) be evidence based, and (7) be used to demonstrate a positive impact on key school objectives. *School values* highlight six items, including (1) whole child enrichment, (2) school physical literacy importance relative to other values, (3) opportunities for life skills to be developed, (4) being embedded throughout school, (5) physical activity being seen as a tool for teaching and learning, (6) the connection between PESSPA and health and wellbeing.

### Strategic agenda

*Strategic agenda* referenced the importance of priorities, within a school, locally and nationally. Four components were identified. *Senior leadership team priorities* focused on individual school’s SLT prioritisation of PESSPA provision and use of WSAs. *Multi-academy trust priorities* focused on the need to align a school’s values with the trust, impacting relative prioritisation of WSAs to PESSPA within school. *Local authority prioritisation* highlighted the importance of their influence over a school’s prioritisation of a WSA to PESSPA, similar, but independent to a multi-academy trust. Finally, *national priorities* were highlighted to have an overarching influence on the prioritisation, or not, of WSAs to PESSPA due to the need for a school to align with national strategies, however did not take into consideration, local context.

### Interpreting the framework

In relation to the TDF, potential key TDF domains are emphasised through the data (Table 3). Of the 14 TDF domains, seven were highlighted in this study, including knowledge, skills, social / professional role and identity, beliefs about capability, intentions, goals, and social influences. In addition, Table 4 finds SLT team values and ethos, cultural barriers and facilitators, and SLT and PE lead quality as potentially important elements of the PIF, as voted for by participants in stage two focus groups and interviews.

## Discussion

### Key findings

This study extends the knowledge surrounding the wide range of influences for the adoption, or not, of WSAs to PESSPA within UK primary schools, providing a framework which supports previous literature and offers unique insights to WSAs previously created, (e.g., PESSPA Toolkit) due to the mapping and emphasis on broader influences of system level determinants (e.g., national priorities) and proposing further evidence for interlinking influences between layers (e.g., local authority influence on SLT priorities). The PIF suggests a multi-layered approach is required for the adoption of a WSA to PESSPA, corroborating previous frameworks, however, differs from earlier findings which concentrate on suggesting modifiable behaviours within a school’s context, to emphasise the focus on a wider breadth and scope of the system.

The PIF relates its layers, constructs, and items back to an individual’s capability, opportunity and motivation to act on a WSA to PESSPA, reflecting other widely accepted health models, such as the socioecological model. The PIF provides a structured representation of how study results extend the typical application of COM-B, and demonstrate the interaction between an individual’s behaviour and meso/macro influences across multiple system layers. In addition, the PIF demonstrates emerging impactful elements (i.e., SLT, school values, knowledgeable PE lead, inter/intra school relationships, local and national governance, and broader school strategy (e.g., child health and wellbeing)) and how they cascade and influence other factors (e.g., schoolteacher buy in) in one succinct framework. The development of the PIF offers a system-orientated conceptualisation of the varied, both in total number and system level, influences of school-staff behaviour within the education sector. TDF analysis has an important bearing on potential intervention design, as it extends the PIFs diagnostic capability, to potential use as a strategic tool to identify meaningful intervention design characteristics across varying system levels. A common primary school intervention is to purchase third party tools (e.g., imoves) which aid teacher PESSPA provision. TDF analysis emphasises the importance of other system level changes which may also be required to create intended behaviour change (e.g., SLT buy in and local authority priorities). Current study results reveal early evidence for targeted strategies towards key influences over the implementation of a WSA to PESSPA highlighted within the PIF, such as leadership team values and ethos, which could be pivotal for widespread adoption of WSAs to PESSPA in UK primary schools. TDF analysis revealed that previous intervention approaches (e.g., teacher training) aimed at influencing PESSPA provision, could have misidentified solutions or overlooked important meso/macro determinants of behaviour which could bolster intervention impact. Moreover, the PIF recognises that policy and key organisations (e.g., Office for standards in education, children services and skills (Ofsted)) likely play a significant and indirect role in the adoption of WSAs to PESSPA, beyond what is typically focused upon (e.g., organisation level intervention). Lastly, the value research and organisations have in the creation of WSAs could also play an integral part in their uptake, providing demonstration for utility, purpose, and benefit to SLT.

### Interpretation of findings

The development of the PIF was motivated by the need to develop a deeper understanding of the vast influences and intersecting relationships over the adoption, or not, of WSAs to PESSPA in UK primary schools. At the centre of the PIF is the behavioural model, COM-B [45], which reinforces the importance of an individual’s influence to implement, or not, a WSA to PESSPA. The PIF however, emphasises that an individual’s ability to implement a WSA to PESSPA, is influenced by many, intersecting micro-meso-macro influences, from personal motivation or confidence, to sufficient resourcing and finance, to national agenda, a finding which is also supported by the 2025 WHO WSA to PESSPA position paper [26]. The PIF emphasises the importance of local mechanisms (e.g., knowledgeable PE lead) and what might be required to achieve global aims (i.e., school culture), through the integration of systems components (i.e., trust) into the model. Current findings also put emphasis on macro (i.e., beyond the school) level influences over individual behaviours for implementing a WSA to PESSPA, such as national and local priorities being intertwined to shape organisational decisions on more granular mechanisms (i.e., teacher training, or financial support). SLT values and ethos were continually highlighted as critical to the adoption of a WSA to PESSPA, alongside the appointment of a knowledgeable PE lead to aid the realisation of a SLT WSA to PESSPA vision. As previously highlighted, SLTs understand the importance of PESSPA [21], and acknowledgement of evidence supporting the positive impact PESSPA has on a child’s physical and mental health, and educational outcomes [6–11], however often struggle to prioritise PESSPA in amongst other key school priorities.

The PIF emphasises other core principles, such as trust (between staff), alignment in goal, and mindset of school (e.g., bold PESSPA provision) as critical for the whole-school adoption of PESSPA initiatives, but recognise the origin in SLT values and ethos. SLT were guided by strategic layers (e.g., national and local authority priorities), and the influence these have on the decision to adopt a WSA to PESSPA or not, and to what degree. Importantly, the alignment of all strategic areas is highlighted, thus the importance of future national and local policy to emphasise the importance of such evidence-based approaches is essential. WHO’s recent recommendations to embed WSA to PESSPA into whole-child-enrichment is reinforced in this study, but perhaps requires greater understanding of how national and local authority policy can influence SLT decision making.

A school’s culture was emphasised, with wider school staff’s perceived relevance and understanding of a WSA to PESSPA being stressed. Systems level thinking, i.e., a collective approach for uniting multiple-stakeholders of a system (e.g., a school) towards a common goal, has been highlighted as difficult to conceptually understand, and perhaps even more difficult to realise in action [51]. It was highlighted that a WSA to PESSPA must demonstrate its link between PESSPA provision and key school priorities, such as attainment, education, or behaviour, to optimise its chances of being adopted, as this enabled more stakeholders within a school to buy into and understand the shared vision. The PIF, therefore may also be a useful tool for WSA to PESSPA design, as it demonstrates and reinforces the variety of barriers, facilitators and motives that impact the utilisation of a WSA to PESSPA within a school.

A strength of the PIF is its multi-layered representation of the influences over the adoption of a WSA to PESSPA. In addition to the cultural and leadership influences over the implementation of a WSA to PESSPA, the PIF highlight the many day-to-day barriers and facilitators. Some of which have been cited previously [19, 20, 22, 31], however not in such a comprehensive framework. The PIF enables relevant professionals in the education sector to understand the array of potential levers that could be used to create change, such as, influencing SLT prioritisation through local authority prioritisation. A key interpretation of the PIF is the depth of relevant intertwined multi-layered influences over the adoption of WSAs, thus, strategic coordination and understanding of school need is required to implement to good effect, any WSA to PESSPA. A potential barrier to the use of the PIF is that it describes many influences (i.e., it may feel overwhelming) over the adoption of a WSA to PESSPA. Current analysis provides early insight however, into potential key influences over the implementation or not, of a WSA to PESSPA within UK primary schools. It must be stated that further research is required to corroborate causal and influencing factors to strengthen the PIF. Previous literature highlights the importance of a considered and stepped approach when thinking about implementation of WSAs, thus this also needs considering when investigating causal mechanisms [31, 52].

### Implications for practice

The presentation of the PIF positions that each framework layer is interconnected, and that the implementation of a WSA to PESSPA require a multi-layered approach, as emphasised in the array of potentially crucial constructs/TDF domains (Tables 2 and 3). A WSA is rooted in this philosophy [25, 37, 53], thus, the creation and analysis of the PIF in relation to the TDF, supports early evidence for the necessity of modern intervention design to include a multi-layered approach, such that it is not only about PESSPA provision, but the leadership, ethos and culture of a school, including staff inter and intra relationships and trust between all staff.

The use of the TDF allowed authors to extract potentially key elements for future intervention focus. Similar themes emerged across the data; emphasising the importance of leadership, the quality and extent of relationships (intra and inter-school), and how these interacted with the school values (i.e., PESSPA WSA relevance). These results support previous literature and progress’ thinking [21, 40] beyond the importance of believing in PESSPA relevance, to demonstration of SLT buy-in through deeply integrated leadership style and quality, manifesting in financial and cultural backing of a WSA to PESSPA. This raises the question however, what is the likelihood of a WSA to PESSPA to be implemented where SLT buy in for PESSPA is low? Also, it does not address previously highlighted issues, where SLT have highlighted their awareness of PESSPA importance, however are pulled in other priority directions. Thus, co-production (i.e., testing of the PIF beyond this co-design process in real world contexts to corroborate causal mechanisms) of the PIF would benefit the literature. We postulate the importance of national and local priorities (strategic agenda) to influence MAT and SLT priorities for WSAs to PESSPA, as this may address the need to, or how to, address school specific context, a known influence of strategic priority [25, 38, 39]. Furthermore, the appointment of a knowledgeable PE Lead (a likely outcome of SLT values) who is able to translate and influence many of the other constructs and items in the PIF seems vital. Additionally, study data, relayed over the TDF, presented potential opportunities for future intervention focus, including the importance of social influences, intentions, and goal setting and school aims. Thus, to impact the implementation of a WSA to PESSPA, school leaders need to consider the importance of shared leadership, likely with the support of a knowledgeable PE lead, how it prioritises PESSPA in the context of other school aims and ambitions, and how it demonstrates the prioritisation of PESSPA in the cultural and physical environments.

### Future research and limitations

Future research should look to validate the PIF through implementation across varying contexts, initiating a phase of co-production to add further framework rigour and robustness due to the small sample size of participants and single region of recruitment within the UK. It would also be beneficial to consider how key TDF domains highlighted could be targeted to create change in enabling WSAs to PESSPA to be adopted in education, namely the importance of leadership and alignment across the organisation towards common and clear goals. This would benefit through the utilisation of the BCW [45]. Results also demonstrate the array of factors that influence a WSA to PESSPA in education, researchers should thus, investigate how schools enact a WSA in a sustainable manner to create change in the areas identified as important, using TDF domains previously mentioned.

Study results should be considered not without limitation. Firstly, although incorporating multiple and relevant stakeholders, these results are from a small, and thus potentially bias sample. Researchers had some prior professional relationships with participants and disciplinary backgrounds in education and public health, which may have influenced data generation and interpretation, shaping participant responses and how meaning was constructed. However, reflexive practices, including team discussion and critical engagement with alternative interpretations, were used to mitigate potential bias and enhance analytical transparency. Future research should, therefore, look to corroborate this data with a larger sample size. Secondly, all participants came from one geographical location, and thus results may be limited to local context. Gaining additional data from a wider variety of rural and urban locations across the UK or abroad would benefit the strength of the PIF.

## Conclusions

The PIF provides an initial, theory-informed framework which highlights a broad overview of the micro-meso-macro influences (i.e., barriers, enablers, motives) that impact the implementation of a WSA to PESSPA in UK primary schools, complementing current literature. The PIF emphasises the importance of a multi-layered systems approach, working concurrently across PIF layers, to tackle physical inactivity in UK primary schools through the adoption of WSAs to PESSPA. The analysis of results in relation to the TDF emphasises a need for future intervention design to focus on factors up-stream of local implementation, and how such interventions cascade down to a local level for the adoption, or not, of a WSA to PESSPA. Thus, to address the question, how are more primary schools supported to adopt a WSA to PESSPA, key influences across the education system need to work collaboratively to address how they are, or could, influence the adoption of a WSA to PESSPA, which future research should aim to address.

## Supplementary Information

Supplementary Material 1.

## Data Availability

Relevant raw materials, such as pictures of flip chart paper with post-it notes from workshops, and anonymised transcripts can be made available upon authors discretion. Data will be made available under the CC-BY licence and stored in Sheffield Hallam University’s Research Data Archive (SHURDA).

## Abbreviations

BCW: Behaviour Change Wheel
COM-B: Capability, Opportunity, Motivation, Behaviour
COREQ: Consolidated criteria for reporting qualitative research
CYP: Children and Young People
MVPA: Moderate to Vigorous Physical Activity
PA: Physical Activity
PE lead: Physical Education Lead
PESSPA: Physical Education, School Sport, Physical Activity
PIF: PESSPA Improvement Framework
SLT: Senior Leadership Team
TDF: Theoretical Domains Framework
UK: United Kingdom
WHO: World Health Organisation
WSA: Whole School Approach
