# Supplementary material for: Co-designing a framework of the influences for the implementation of whole school approaches to Physical Education, School Sport and Physical Activity (PESSPA) in UK primary schools: development of the PESSPA Improvement Framework (PIF)
Source: BMC Public Health. 2026 Aug 7;26:3194. doi: 10.1186/s12889-026-28832-w (PMC13732920; doi:10.1186/s12889-026-28832-w)
Supplement: Supplementary file 1 [file 12889_2026_28832_MOESM1_ESM.docx]

**Topic guides and questionnaire outline**

**Topic guide for workshop 1 / interviews**

| **Theme** | **Question** |
| --- | --- |
| Ideal of WSA to PA in practice | “*All members of the school community (school leaders, middle management, teaching and non-teaching staff, learners, parents and families) feel responsible and play an active role in tackling physical inactivity*.” (European Commission)    What does this look like – blue sky thinking? |
| Generation of ideas | Jane is a primary school teacher who recently moved to a school with no WSA to PA. What might Jane encounter when trying to implement a WSA to PA?  What are the root causes of these?  What are the **Contextual features** that influence the implementation of a WSA to PA in primary schools?   - The context in which the school sits in (internal and external environments)   What are the **mechanisms** that aid the elicitation of change for the implementation of a WSA to PA in primary schools?   - Physical levers that influence...   What are the **values** or working that will enable the implementation of a WSA to PA in primary schools?   - Core beliefs of the school and individual. |
| Prioritisation of ideas | What are the most significant hurdles Jane will need to overcome to be able to begin to implement a WSA to PA in her new primary school?  What are the most significant facilitators Jane will need to overcome to be able to begin to implement a WSA to PA in her new primary school?  Prioritise these in groups of impact (most, middle, least) of influence over implementation of a WSA to PA in primary schools.   - As one list |
| Further development of ideas | Regarding Context and Mechanisms, are there sub-categories   - E.g., in school, political, external   Anything missing? Either a category or individual factor from within a category? |

**Topic guide for workshop 2 / interviews**

| **Theme** | **Question** |
| --- | --- |
| Workshop 1 raw themes and codes | Presentation of raw themes and codes in an initial framework |
| Current framework | How well might the items, themes and feel of the framework, represent what Jane may encounter when trying to implement a WSA to PA in reality?  Discuss if you think the crucial factors that Jane may encounter are highlighted.  How well do these themes represent reality?  How well do these themes explain the barriers and facilitators to implementing a WSA to PA?  What is missing, if anything (individual codes)? |
| Framework evolution | What connects or links these individual codes?  How is a WSA to PA operationalised?  What stable structures are helpful (e.g., meetings / training)?  What are the hidden influences that affect the implementation of a WSA to PA? |
| Critical components | Discuss what are the most significant (top 3) influences that will influence Jane in her quest to implement a WSA to PA in her new school. |

**Final Questionnaire**

| **Question** | **Potential responses** |
| --- | --- |
| How well do you feel the current PESSPA Improvement Framework Diagram reflects the overarching Themes and Constructs that influence the implementation of a whole school approach to physical activity in primary schools | Not well at all  Slightly well  Moderately well  Very well  Extremely well |
| If there are any 'Themes' or 'Constructs' of the diagram that you do not agree with, think need moving or rewording, or if there are 'Themes' or 'Constructs' you believe are missing and need adding, please can you highlight/detail all of these here | Open text |
| How well do you feel the current PESSPA Improvement Framework Table reflects the specific (e.g., labelled 'Items' in the Table) Items that influence the implementation of a whole school approach to physical activity in primary schools | Not well at all  Slightly well  Moderately well  Very well  Extremely well |
| If there are any 'Items' within the Table that you do not agree with, think need moving or re-wording, or if there are 'Items' you believe are missing and need adding, please can you highlight/detail all of these here | Open text |
| If there are any 'Items' within the Table that you do not agree with, think need moving or re-wording, or if there are 'Items' you believe are missing and need adding, please can you highlight/detail all of these here | Open text |
| Please can you provide any final thoughts and opinions of the PESSPA Improvement Framework here | Open text |
